# The effect of Ramadan intermittent fasting on anthropometric, hormonal, metabolic, inflammatory, and oxidative stress markers in pre-and post-menopausal women: a prospective cohort of Saudi women

**DOI:** 10.3389/fnut.2024.1437169

**Published:** 2024-12-04

**Authors:** Nada A. Al Zunaidy, Abdulrahman S. Al-Khalifa, Maha H. Alhussain, Sami A. Althwab, Mohammed A. Mohammed, MoezAlIslam E. Faris

**Affiliations:** ^1^Department of Food Science and Human Nutrition, College of Agriculture and Food, Qassim University, Buraydah, Saudi Arabia; ^2^Department of Food Science and Nutrition, College of Food and Agricultural Sciences, King Saud University, Riyadh, Saudi Arabia; ^3^Department of Clinical Nutrition and Dietetics, Faculty of Allied Medical Sciences, Applied Science Private University, Amman, Jordan

**Keywords:** intermittent fasting, diet, anthropometric, cytokines, oxidative stress, antioxidant, menopausal

## Abstract

**Background:**

The menopausal transition significantly affects cardiometabolic health, primarily due to changes in reproductive hormones, particularly decreased estrogen levels and relative androgen excess. Adult Muslim women, both pre-and post-menopausal, are mandated to observe Ramadan intermittent fasting (RIF) every year. Therefore, the current study was designed to investigate RIF’s effects on pre-menopausal (PRE-M) and post-menopausal (POST-M) healthy women’s cardiometabolic health markers. This study further evaluated the relationship between tested markers and the participant’s basic variables, such as BMI and body fatness. Due to differences in physiological and metabolic biomarkers between groups, RIF is likely to impact PRE-M and POST-M women differently.

**Methods:**

This study included 62 healthy women (31 PRE-M, aged 21–42 years, and 31 POST-M, aged 43–68 years) who observed RIF. Anthropometrics, sex hormones, lipid profile, pro-inflammatory (TNF-*α*), anti-inflammatory (IL-10) cytokines, the oxidative stress markers malondialdehyde (MDA), total antioxidant capacity (TAC), superoxide dismutase (SOD), glutathione peroxidase (GPx), and aging biomarker insulin-like growth factor-1 (IGF-1); all were tested 1 week before and at the fourth week of Ramadan.

**Results:**

Body weight, BMI, waist circumference, body fat percentage (BFP), fat mass, fat mass index, triglycerides, and diastolic blood pressure significantly (*p* < 0.05) decreased at the end of Ramadan in both groups in comparison to the pre-fasting period. Contrarily, HDL, SOD, GPx, and IL-10 significantly (*p* < 0.05) increased in both groups. Estrogen levels significantly (*p* < 0.05) decreased in PRE-M women, whereas significantly (*p* < 0.05) increased in POST-M women. The progesterone levels, TAC, MDA, and IGF-1 remained unchanged in both groups. TNF-*α* significantly decreased in both groups, but the magnitude of reduction was higher in PRE-M women. Sex hormones and some metabolic biomarkers, especially in POST-M women, variably exhibited positive or negative relationships to BMI and BFP. RIF may influence the levels of estrogen, TNF-*α*, and IL-10 through improvements in metabolic health, reductions in body fat, activation of autophagy, modulation of immune responses, and changes in hormonal regulation.

**Conclusion:**

The RIF was generally associated with improved anthropometric, metabolic, inflammatory, and oxidative stress markers in both PRE-M and POST-M healthy women. Adhering to healthy dietary and lifestyle guidelines by pre-and post-menopausal women during Ramadan may foster the health benefits gained.

## Introduction

The transition from pre-menopausal (PRE-M) to post-menopausal (POST-M) phase is associated with drastic changes in many hormonal and metabolic markers. After menopause, women exhibit a sustained increase in hemodynamic load and altered sympathetic nervous system activity that may contribute to pathological functional and structural modifications in the blood vessels and heart (1). Modifications in estradiol and progesterone levels may cause changes during the PRE-M phase (2). Some studies (3, 4) revealed inconsistent results regarding the effects of IF and RIF on reproductive hormones in healthy women. According to Ko and Kim (5), due to hormonal changes during the menopausal transition period, such as decreased estrogen levels and increased levels of circulating androgens, a variety of lipid metabolic disorders emerge. These disorders may cause metabolic syndromes, including cardiovascular diseases and type 2 diabetes. They also stated that lipid metabolism abnormalities have an impact on body fat mass, fat-free mass, fatty acid metabolism, and many elements of energy metabolism, such as basal metabolic ratio, adiposity, and obesity. Menopause is also connected with changes in the amounts of various lipids in the blood, which causes lipid peroxidation and the development of insulin resistance, abdominal obesity, and dyslipidemia (5). According to El Khoudary et al. (6), the menopause transition has a substantial impact on cardiovascular health and results in a variety of lifestyle and dietary modifications. Furthermore, they discovered different patterns of sex hormone alterations and negative changes in body composition, lipoproteins, lipids, and vascular health during menopause. These modifications raise the likelihood of acquiring POST-M illnesses.

Various physiological effects and positive findings have been reported for caloric restriction and intermittent fasting (IF) in men and rodents on several disease conditions, including prolonged lifespan, decreased aging and its neurological diseases, cardiovascular diseases, and cancer-related mortalities, enhanced insulin sensitivity, and reduced inflammation, and oxidative stress (7–11). IF can affect energy signaling pathways that are regulated by cAMP-responsive element binding protein (CREB) and AMP-activated protein kinase (AMPK), as well as the pro-growth mTOR pathways and the expression of circadian clock genes. The fasting-sensitive AMPK is activated by elevated AMP levels, which can subsequently influence the circadian clock by modulating essential circadian regulators (12).

Ramadan fasting is a holy month for Muslims during which all adult healthy Muslims are mandated to abstain from food, drink (including water), and sexual activities from dawn to sunset. Thus, the month of Ramadan is associated with drastic lifestyle and dietary modifications. The main aspects of modification include changing food consumption patterns (13, 14), reduced physical activities (15), and night sleep periods with increased sleep intervals during the day (16). Considering that people alternate between fasting and feasting within 24 h, Ramadan fasting is a typical form of IF, and it is regarded as the most commonly and globally observed religious form of IF (17).

Epidemiological studies have established the beneficial impacts of observing Ramadan intermittent fasting (RIF), including body weight reduction (18), reduced body fat (19, 20), especially visceral fat (19), normalizing glucometabolic regulation (20), cardiometabolic (21), and liver functioning (22), accompanied by metabolomic (23), lipidomic (24), and microbiomic alterations (25). Improvements in metabolic health (21), reductions in body fat (26), activation of autophagy (27), modulation of immune responses (28), and changes in hormonal regulation (29, 30) are among the proposed mechanisms underpinning the reported health effects of observing RIF.

Sex, presented in terms of the body’s sex hormones, directly impacts metabolic reactions in both males and females, and determines the magnitude of changes in anthropometric, metabolic, and physiological aspects, as well as dietary changes upon the observance of RIF in both healthy and disease conditions. This emphasizes the role of sex as one of the determinant factors that affect the health outcomes of observing RIF (31, 32) and necessitates examining RIF impacts on the two sexes separately.

Observing RIF presents unique health implications for both pre-and post-menopausal women. For pre-menopausal women, hormonal fluctuations may be affected by IF, potentially leading to altered menstrual patterns (33). During Ramadan, weight management can become a challenge, as fasting may result in weight loss or gain depending on dietary choices during non-fasting hours (13, 18). Improved insulin sensitivity and improved lipid profiles are possible benefits of observing RIF (20, 21, 34); however, there is a risk of hypoglycemia for those with metabolic conditions (35). For menopausal women, the effects of fasting can vary significantly due to decreased estrogen levels, which can lead to symptoms like hot flashes (36). Additionally, menopausal women need to consider bone health, as adequate calcium and vitamin D intake is crucial during Ramadan to counter osteoporosis risk (37, 38). Fasting, including RIF, can also improve cardiovascular health and reduce inflammation, benefiting menopausal women who may suffer from related conditions (21, 39, 40).

Considering the vast metabolic and hormonal effects of practicing fasting in general, and RIF in particular, it becomes rationalized and necessary to examine the effect of the observance of RIF by pre-and post-menopausal Saudi women and to elaborate how different health markers change before and during the fasting days of Ramadan. Therefore, this study aimed to assess the effects of RIF on PRE-M and POST-M women’s anthropometric indices, lipid profiles, and metabolic biomarkers. This study further evaluated the relationship between the tested biomarkers and participant variables, including age, body fat, and BMI.

## Materials and methods

### Study design and subjects

The current study used an open label, longitudinal, follow-up design with convenience sampling and pre-and post-test data collection, as described elsewhere (41, 42). This study was conducted during Ramadan, from the first of April to the first of May 2022. An information sheet was distributed to the people who met the eligibility requirements. This study’s enrollment was advertised on social media, in hospital bulletins, and personal correspondence.

The data was gathered twice: once a week before Ramadan (R1) and again at the end of Ramadan’s third week, after 21–29 days of fasting (R2). The participants in Ramadan observed fasting from dawn to sunset (about 14 h), with an average fasting hour of 350 h for the two groups. The participants in this study were not given any special dietary guidelines and were instructed to eat and exercise as normal before Ramadan. The size of the sampling was set based on the *A priori* power analysis, which was conducted using G*Power 3.1. software (43). The analysis indicated that a sample of 30 for each group, a total of 60 participants, would allow 80% power to detect a medium effect size (*ƒ* = 0.50, *𝑎* = 0.05), and the participants were selected using inclusion and exclusion criteria. Through social media, we conveniently recruited 62 women aged 21–68 from Saudi Arabia’s Qassim (Unaiza area). We contacted all those interested in this study and requested that they attend an official meeting at King Saud Hospital, Unaiza. In this meeting, the participants were briefed about the study objectives and protocol, their eligibility and medical status were assessed, and they were given the opportunity to sign an informed consent form. The bodily measurements and blood samples were taken between 11 a.m. and 1 p.m. during both visits of the two time points.

**Figure 1:**
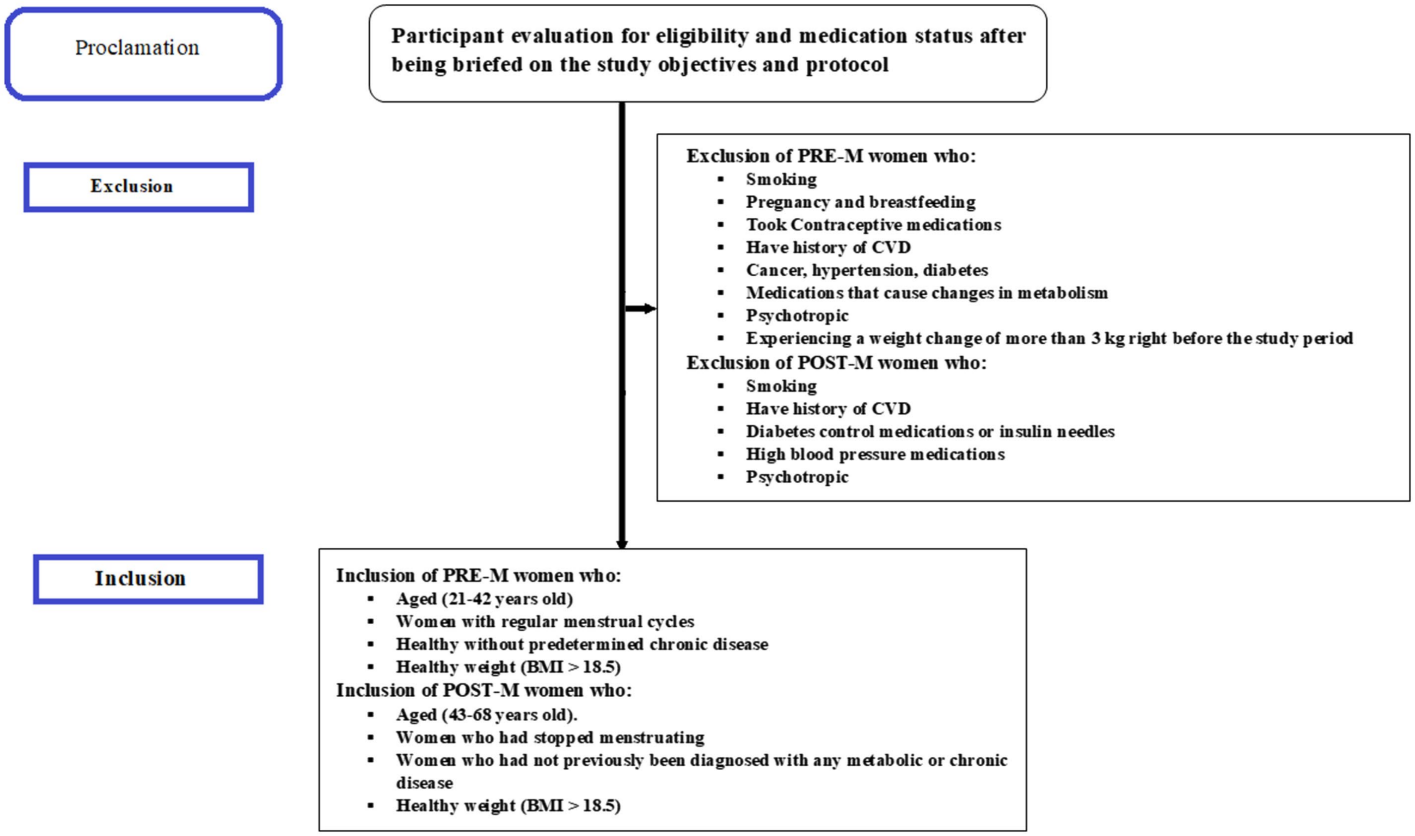
Participant recruitment flow chart.

Before the study started, all subjects provided written informed consent. According to the questionnaire and clinical data, those who responded were divided into two groups: PRE-M women (21–42 years old) and POST-M women (43–68 years old). Healthy women with regular menstrual cycles were clinically defined as PRE-M, while those who had stopped menstruating at least a year before sample collection were classified as POST-M; both categories were then considered. Those with a record of smoking, CVD, pregnancy, cancer, hypertension, breastfeeding, diabetes, and even non-diabetics but taking medicines for sugar control, as well as those taking medications that cause changes in metabolism such as antiretrovirals, corticosteroids, antiseizure, psychotropic, insulin, sulfonylurea, and thiazolidinediones, or experiencing a weight change of more than 3 kg right before the study period, were excluded. Inclusion and exclusion criteria applied are depicted in Figure 1.

### Anthropometric measurement

Anthropometric parameters were measured in two visits (triplicate in each visit) using the body composition analyzer ACCUNIQ BC360 (SELVAS Healthcare Inc., Daejeon, Korea). The reproducibility and validity for body composition measuring BIA device in adult women is reported elsewhere (44). Electrodes were placed on the respondent’s hands and feet in the standing position. The device was connected to an Ultrasonic Height meter, which automatically measured the body weight, body mass index (BMI), hip circumference (HC), waist circumference (WC), waist-to-height ratio (WHtR), and waist-to-hip ratio (WHR), fat mass (FM), and fat-free mass (FFM). The participants fasted for 4 h before testing, and their palms and soles were cleaned after each measurement. Fat mass index (FMI, kg/m^2^) was calculated by dividing the fat mass (FM, kg) by height square (m^2^), whereas the fat-free mass index (FFMI, kg/m^2^) was calculated by dividing the fat-free mass (FFM, kg) by height square (m^2^).

### Blood sample collection and analysis

Blood samples were collected before and after Ramadan at the two time points. A venous blood sample (10 mL) was collected from each subject after at least 8 h of fasting. To eliminate the impact of timing and dietary intake on the measured biochemical parameters and to guarantee consistent fasting duration at both time points, the samples were taken between 11 a.m. and 1 p.m. during both visits. The blood was centrifuged for 15 min at 3000 rpm within an hour of collection. The serum was coded and kept at-80°C until utilized for biochemical analysis. The blood samples were analyzed in the laboratory of KSH, Unaizah (Qassim district), and the laboratory of Qassim University. ELISA kits were used to compare the required measurements to a specific standard for each parameter. Initially, the standard of each parameter was tested, and related regression and trend lines were plotted. Then, the relevant parameters from the blood samples were calculated accordingly. The kits were precisely handled by following the instructions to perform multiple replicates for each parameter.

### Assessment of blood pressure

Following each interview (from baseline to end of research), blood pressure was recorded in the left arm (mmHg) using a standard mercury sphygmomanometer with an appropriate cuff size. Participants with an empty bladder were seated after resting for at least 10 min in a quiet area.

### Cytokines assay

ELISA-based techniques were adopted to analyze the cytokines quantitatively. Tumor necrosis factor-*α* (TNF-*α*) (Cat. No E0082Hu ELISA Kit, Shanghai, China) was measured as a pro-inflammatory biomarker. A standard curve was constructed using the standard solution in the kit. Interleukin (IL)-10 (Cat. No. E0102Hu, IL10 ELISA Kit, Shanghai, China) was measured as an anti-inflammatory biomarker and a standard curve was created using the standard solution.

### Determination of oxidative stress biomarkers

ELISA kit (Cat. No. E2199Hu) was used to assess the Total antioxidant capacity (TAC) in samples. The absorbance of the final solution was measured at 450 nm, and a standard solution was used to generate a standard curve. A colorimetric assay kit (Cat. No E1371Hu, Shanghai, China) was used to calculate the lipid peroxidation as malondialdehyde (MDA) by measuring thiobarbituric acid reactive substance (TBARS). The standard curve was constructed using the standard solution in the kit. Superoxide dismutase (SOD) activity was determined using a SOD assay kit (Cat. No E0918Hu, Shanghai, China), whereas ELISA kits (Cat. No E3696Hu) were employed to assess the Glutathione peroxidase (GPx) activity. The standard curve was created using the standard solution. ELISA kit (Cat. No. E0103Hu) was used to measure the insulin-like growth factor-1 (IGF-1), and a standard curve was generated from the standard solution, all done in the laboratory of Qassim University.

### C-reactive protein detection

The C-reactive protein (CRP) was tested in the laboratory of King Saud Hospital (KSH) using a qualitative CRP analyzing kit (BioTime, Xiamen, Fujian, China).

### Blood glucose, lipid profile, and sex hormone determination

Blood glucose levels and lipid profile (total cholesterol (TC), high-density lipoprotein cholesterol (HDL-C), low-density lipoprotein cholesterol (LDL-C), and triglyceride (TG)) were determined using colorimetric kits and a Dimension Xpand Plus Chemistry Analyzer. Progesterone and estrogen were measured using XN1000-Cobas E411 (Sysmex-Rauch-Hitachi, Tokyo, Japan) in KSH.

### Statistical analysis

Statistical analyses were carried out using SPSS software (version 25) (SPSS Inc., Chicago, IL, USA). Repeated measures of ANOVA were applied to study the correlation within and between experimental groups’ data (anthropometric, blood glucose, lipid profile, blood pressure, and sex hormones levels as well as circulating levels of oxidative stress, aging, and inflammatory biomarkers) along with the time (before and at the end of Ramadan) of the measurements (time point 1, time point 2, etc.). Similar to an ANOVA, time is treated as a categorical variable rather than a continuous variable in repeated measures ANOVA. Spearman correlation coefficients and simple regression analysis were used to determine the relationship between anthropometric indices and age as independent variables and sex hormones, circulating levels of oxidative stress, aging, and inflammatory biomarkers as dependent variables for PRE-M and POST-M healthy women.

## Results

### Changes in anthropometric indices, lipid profile, and sex hormone levels

Table 1 presents the anthropometric, basic hematological, and hormone levels in PRE-M and POST-M women before and at the end of RIF’s period. The parameters, including weight, BMI, waist circumferences, the ratio of waist-to-height, PBF, FM, FMI, and TG, significantly decreased in PRE-M and POST-M women at the end of the fasting period (main effect of time, *p* < 0.001, or *p* = 0.002) without significant differences between the two groups. Contrarily, HDL-C levels significantly increased in PRE-M (1.62 to 4.75 mmol/L) and POST-M (1.40 to 2.56 mmol/L) women (main effect of time, *p* = 0.027); however, the difference between R1 and R2 was significant. The women belonging to PRE-M experienced greater increases in HDL-C levels compared to the POST-M group of women (group × time interaction, *p* = 0.008). Diastolic blood pressure was significantly increased in the PRE-M group, whereas it decreased in the POST-M group (main effect of time, *p* < 0.001); however, the difference between R1 and R2 in the POST-M was highly significant. The effect was higher in the POST-M women group as compared to the PRE-M women group (group × time interaction, *p* = 0.021). Estrogen also significantly decreased in the PRE-M group and increased in the POST-M group (main effect of time, *p* = 0.046); however, the difference between R1 and R2 in terms of estrogen in the PRE-M was highly significant. The change was greater in the PRE-M group than in the POST-M group of women (group × time interaction, *p* = 0.01). However, the parameters such as hip circumference, waist-to-hip ratio, total body water, total cholesterol, progesterone level, FFM, LDL-C, systolic blood pressure, and fasting glucose did not exhibit significant changes between and within groups.

**Table 1:** Changes in anthropometric, blood glucose, lipid profile, blood pressure, and sex hormone levels before (R1) and at the end of Ramadan (R2) for PRE-M and POST-M healthy women (*n* = 62).

Parameter	PRE-M (*n* = 31)		Difference*	POST-M (*n* = 31)		Difference*	*p*-value time	*p*-value group × Time
	R1	R2		R1	R2			
	Mean ± SD	Mean ± SD		Mean ± SD	Mean ± SD			
Weight (kg)	61.33 ± 11.73	59.42 ± 11.38	−1.91	69.08 ± 11.26	66.97 ± 11.28	−2.11	< 0.001	0.628
Body mass index (BMI) (kg/m^2^)	24.45 ± 4.56	23.71 ± 4.49	−0.74	28.57 ± 3.83	27.43 ± 4.00	−1.14	< 0.001	0.129
Waist circumference (cm)	72.74 ± 9.17	68.90 ± 9.13	−3.84	86.63 ± 9.42	81.00 ± 8.50	−5.63	< 0.001	0.085
Hip circumference (cm)	99.71 ± 11.74	97.87 ± 7.94	−1.84	102.19 ± 18.99	98.69 ± 12.89	−3.5	0.168	0.664
Waist-to-height ratio (WHtR) (cm)	0.46 ± 0.06	0.44 ± 0.06	−0.02	0.56 ± 0.07	0.52 ± 0.06	−0.04	< 0.001	0.068
Waist-to-hip ratio (WHR) (cm)	0.73 ± 0.06	0.70 ± 0.05	−0.03	1.05 ± 1.30	0.84 ± 0.16	−0.21	0.618	0.187
Total body water (kg)	31.29 ± 3.13	30.89 ± 2.84	−0.40	30.68 ± 2.87	30.89 ± 3.30	0.21	0.679	0.135
Percent body fat (%)	29.62 ± 8.72	28.72 ± 8.47	−0.90	38.56 ± 5.43	37.18 ± 5.52	−1.38	< 0.001	0.293
Fat mass (FM)(kg)	19.08 ± 8.61	18.17 ± 8.27	−0.91	27.16 ± 7.82	25.76 ± 7.41	−1.40	< 0.001	0.191
Fat-free mass (FFM) kg	42.30 ± 3.94	42.16 ± 3.87	−0.14	41.93 ± 3.94	42.20 ± 4.52	0.27	0.661	0.222
FMI (kg/m^2^)	7.56 ± 3.38	6.88 ± 3.28	−0.68	11.08 ± 2.95	10.52 ± 2.85	−0.56	< 0.001	0.148
FFMI (kg/m^2^)	16.89 ± 1.59	16.83 ± 1.56	−0.06	17.23 ± 1.58	17.32 ± 1.54	0.09	0.796	0.277
LDL-C (mmol/L)	2.49 ± 0.99	2.67 ± 0.90	0.18	3.43 ± 0.89	3.51 ± 0.85	0.08	0.326	0.701
Total cholesterol (mmol/L)	4.49 ± 1.08	4.75 ± 0.69	0.26	5.47 ± 0.86	5.14 ± 1.56	−0.33	0.792	0.086
HDL-C (mmol/L)	1.62 ± 0.28^bc^	4.75 ± 0.35^a^	3.13	1.40 ± 0.43^bc^	2.56 ± 0.39^b^	1.16	0.027	0.008
Fasting glucose (mmol/L)	4.64 ± 1.85	4.06 ± 1.84	−0.58	5.82 ± 2.62	5.45 ± 2.40	−0.37	0.186	0.776
Triglycerides (TG)(mmol/L)	0.81 ± 0.32	0.72 ± 0.50	−0.09	1.16 ± 0.44	0.90 ± 0.51	−0.26	0.002	0.113
Systolic blood pressure (SBP)(mmHg)	110.81 ± 13.50	113.78 ± 8.82	2.97	127.47 ± 19.88	125.91 ± 23.98	−1.56	0.653	0.156
Diastolic blood pressure (DBP) (mmHg)	77.00 ± 12.20^ab^	77.39 ± 7.99^ab^	0.39	81.41 ± 13.24^a^	71.00 ± 10.31^c^	−10.41	< 0.001	0.021
Estrogen (pmol/L)	649.58 ± 95.03^a^	417.13 ± 17.75^b^	−232.45	42.51 ± 6.39^cd^	49.44 ± 3.18^c^	6.93	0.046	0.010
Progesterone (pmol/L)	9.74 ± 1.75	9.81 ± 2.05	0.07	1.27 ± 0.18	0.39 ± 0.07	−0.88	0.878	0.858

Values are expressed as means ± SD. *p*-value: Repeated measures ANOVA with groups (pre-menopausal women, post-menopausal women) as the between-subject factor and time before (R1) and at the end of Ramadan (R2) as the within-subject factor. *Difference = R2 – R1. Means not sharing a common letter are significantly different (group × time interaction). The values with different superscript letters (a,b,c,d) in a column are significantly different (*p* < 0.05).

### Changes in cytokines, oxidative stress biomarkers, and C-reactive protein (CRP)

Table 2 demonstrates the circulating levels of oxidative stress, and inflammatory and anti-inflammatory markers. TAC, MDA, and IGF-1 remained unchanged between and within the two groups during fasting and non-fasting periods. There was a highly significant difference between R1 and R2 with regard to GPx, IL-10, and TNF-*α* in the PRE-M group than in the POST-M group of women. SOD activity significantly increased in both PRE-M and POST-M women groups (main effect of time, *p* = 0.016), and the two groups did not vary significantly. GPx (main effect of time, *p* = 0.05) and IL-10 (main effect of time, *p* = 0.025) increased dramatically in both PRE-M and POST-M women. The rise in GPx (group × time interaction, *p* = 0.045) and IL-10 (group × time interaction, *p* = 0.029) was significantly higher in PRE-M women compared to the POST-M women. Contrarily, TNF-*α* significantly decreased in both groups (main effect of time, *p* = 0.021). The reduction was found to be more significant in PRE-M women (group × time interaction, *p* = 0.048). Table 3 shows the presence or the absence of CRP in PRE-M and POST-M women. The results demonstrated the presence of inflammation in only two (6.45%) women in the PRE-M group, and the number decreased to one by the end of Ramadan. These findings suggest that fasting during the day in Ramadan could counter-elevate inflammatory and oxidative stress markers to some extent. It could further reduce low-grade systemic inflammation, oxidative stress, and related negative health effects in healthy individuals. At the end of Ramadan, however, the number of women in the POST-M group with oxidative stress and inflammation increased from 5 (16.13%) to 10 (32.26%). This may be temporary due to sudden hormonal changes during the month of Ramadan.

**Table 2:** Changes in circulating levels of oxidative stress, aging, and inflammatory biomarkers before (R1) and at the end of Ramadan (R2) for PRE-M and POST-M healthy women (*n* = 62).

Parameter	PRE-M (*n* = 31)			POST-M (*n* = 31)			*p*-value time	*p*-value group × time
	R1	R2	Difference*	R1	R2	Difference*		
	Mean ± SD	Mean ± SD		Mean ± SD	Mean ± SD			
TAC (U/ml)	21.18 ± 5.08	24.40 ± 7.39	3.22	15.66 ± 6.01	18.01 ± 8.50	2.35	0.482	0.922
MDA (nmol/ml)	2.37 ± 9.48	2.31 ± 4.36	−0.06	2.14 ± 6.09	1.61 ± 2.89	−0.53	0.147	0.636
SOD (U/ml)	11.87 ± 1.72	15.96 ± 1.30	4.09	11.13 ± 1.43	13.10 ± 2.86	1.97	0.016	0.334
GPx (g/m^l^)	136.44 ± 10.67^b^	143.86 ± 12.67^a^	7.42	125.09 ± 5.90^c^	126.62 ± 8.51^c^	1.53	0.050	0.045
IGF-1 (ng/ml)	119.24 ± 4.01	119.62 ± 4.38	0.38	100.31 ± 3.84	100.69 ± 4.34	0.38	0.406	0.989
IL-10 (pg/ml)	30.31 ± 3.62 ^b^	39.73 ± 2.19^a^	9.42	21.46 ± 2.98^c^	21.56 ± 1.95^c^	0.10	0.025	0.029
TNF-α (ng/ml)	194.45 ± 8.02^a^	183.30 ± 9.65^b^	−11.15	123.43 ± 9.71^c^	117.19 ± 2.13^c^	−6.24	0.021	0.048

Values are expressed as means ± SD. *p*-value: Repeated measures ANOVA with groups (pre-menopausal women, post-menopausal women) as the between-subject factor and time before (R1) and at the end of Ramadan (R2) as the within-subject factor.

*Difference = R2 – R1. Means not sharing a common letter are significantly different (group × time interaction). TAC, Total antioxidant capacity; MDA, Lipid peroxidation as malondialdehyde; SOD, Super oxide dismutase; GPx, Glutathione peroxidase enzyme; IGF-1, Insulin-like growth factor-1; IL-10, Interleukin-10; TNF-*α*, Tumor necrosis factor-*α*. The values with different superscript letters (a,b,c,d) in a column are significantly different (*p* < 0.05).

**Table 3:** Changes in qualitative C-reactive protein (CRP) before (R1) and at the end of Ramadan (R2) for PRE-M and POST-M healthy women (*n* = 62).

Results	PRE-M (*n* = 31)					POST-M (*n* = 31)				
	R1	%	R2	%	*p*-value	R1	%	R2	%	*p*-value
Positive	2	6.45	1	3.23		5	16.13	10	32.26	
Negative	29	93.55	30	96.77	0.071	26	83.87	21	67.74	0.015
Total	31	100.0	31	100.0		31	100.0	31	100.0	

* *p* ≤ 0.05; ** *p* ≤ 0.01 according to Chi-square test.

### Association of BMI, PBF, and age with cytokines, aging, and oxidative stress biomarkers

Table 4 summarizes the relationship between BMI, percent body fat, and metabolic biomarkers. The findings revealed that the BMI of women in the PRE-M group was not associated with any of the biomarkers at both the testing periods (before and at the end of Ramadan). However, for the same group, BMI had a substantial positive connection with estrogen levels before and after fasting, as well as TNF-*α* before fasting. POST-M women’s BMI had a significant negative correlation with the progesterone levels before and at the end of the fasting period. Similarly, a significant positive correlation of PBF was observed with the estrogen level in POST-M women, whereas it remained negatively correlated with the progesterone level in both groups (PRE-M and POST-M). A significant positive association was noted between PBF and TNF-α in both groups before fasting. The PBF of POST-M women also exhibited a significant positive association with MDA before fasting. A significant negative association was noted between BMI or PBF and IGF-1in both groups at the end of the fasting period. Table 5 presents the association between age and metabolic biomarkers. PRE-M women’s age was significantly (*p* ≤ 0.01) and negatively associated with progesterone level before the fasting period. Moreover, lipid MDA was significantly and negatively correlated with PRE-M age before and at the end of the fasting period. Before Ramadan, there was a positive relationship between IL-10 and age in both groups. However, age was negatively correlated with the IGF-1 marker before and after the fasting period in POST-M women. Other biomarkers were either positively or negatively related to the age of the respondents, but only at a non-significant level.

**Table 4:** Association between body mass index, body fatness, and metabolic biomarkers before (R1) and at the end of Ramadan (R2) for PRE-M and POST-M healthy women (*n* = 62).

Independent variable/ dependent variable												
	PRE-M (*n* = 31)						POST-M (*n* = 31)					
	R1			R2			R1			R2		
	*r*	(*β*, *r*^2^)	*p*-value	*r*	(*β*, *r*^2^)	*p*-value	*r*	(*β*, *r*^2^)	*p*-value	*r*	(*β*, *r*^2^)	*p*-value
Body mass index (BMI)												
Estrogen	0.132	(−0.001, 0.017)	0.478	0.111	(0.028, 0.012)	0.551	0.286*	(0.017*, 0.082)	0.012	0.351*	(0.441*, 0.123)	0.049
Progesterone	−0.216	(−0.003, 0.047)	0.243	−0.111	(−0.028, 0.012)	0.551	−0.152**	(0.006**, 0.023)	0.007	−0.412*	(−0.800*, 0.169)	0.019
TAC	0.040	(0.006, 0.002)	0.829	0.092	(0.007, 0.001)	0.624	−0.026	(−0.008, 0.003)	0.889	−0.047	(−0.010, 0.002)	0.800
MDA	−0.032	(−0.002, 0.001)	0.866	0.125	(0.014, 0.016)	0.502	−0.178	(−0.011, 0.032)	0.330	−0.085	(−0.029, 0.169)	0.644
SOD	0.087	(0.003, 0.008)	0.642	−0.028	(−0.001, 0.001)	0.880	−0.055	(−0.002, 0.003)	0.764	−0.001	(−0.601, 0.0002)	0.994
GPx	0.054	(0.005, 0.003)	0.773	−0.204	(−0.015, 0.042)	0.271	−0.093	(−0.009, 0.009)	0.614	−0.019	(−0.003, 0.0002)	0.918
IL-10	0.059	(0.001, 0.004)	0.751	0.031	(0.001, 0.004)	0.867	−0.058	(−0.001, 0.003)	0.751	−0.047	(−0.001, 0.002)	0.800
TNF-α	0.122	(0.003, 0.015)	0.215	0.022	(0.015, 0.008)	0.906	0.075*	(0.012*,0.006)	0.043	0.040	(0.001, 0.002)	0.828
IGF-1	−0.054	(−0.018, 0.003)	0.773	−0.047*	(−0.015*, 0.002)	0.048	−0.093	(−0.040, 0.009)	0.614	−0.034**	(−0.071**, 0.005)	0.001
Percent body fat (PBF)												
Estrogen	−0.107	(−0.001, 0.011)	0.567	0.077	(0.031, 0.023)	0.679	0.239**	(0.120**, 0.057)	0.007	0.153	(0.092, 0.003)	0.774
Progesterone	−0.214*	(−0.045*, 0.0001)	0.033	−0.077	(−0.037, 0.006)	0.679	−0.127**	(−0.217**, 0.016)	0.004	−0.292	(−0.721, 0.085)	0.104
TAC	0.037	(0.010, 0.001)	0.842	0.084	(0.026, 0.007)	0.655	−0.099	(−0.045, 0.010)	0.591	−0.236	(−0.067, 0.056)	0.194
MDA	0.097	(0.014, 0.009)	0.603	0.151	(0.031, 0.023)	0.416	0.354*	(0.031*, 0.126)	0.047	0.289	(0.134, 0.084)	0.108
SOD	−0.025	(−0.001, 0.001)	0.894	−0.086	(−0.005, 0.007)	0.644	−0.125	(−0.007, 0.016)	0.496	−0.123	(−0.006, 0.015)	0.501
GPx	0.042	(0.007, 0.002)	0.824	−0.219	(−0.029, 0.048)	0.238	−0.228	(−0.030, 0.052)	0.210	−0.165	(−0.032, 0.027)	0.366
IL-10	0.058	(0.001, 0.003)	0.757	0.034	(0.034, 0.001)	0.855	−0.174	(−0.004, 0.030)	0.340	−0.236	(−0.004, 0.056)	0.194
TNF-α	0.039*	(0.021*,0.002)	0.036	0.037	(0.001, 0.001)	0.845	0.142**	(0.005, 0.020)	0.009	−0.179	(0.009, 0.032)	0.326
IGF-1	−0.068	(−0.042, 0.005)	0.716	−0.012*	(−0.007*, 0.002)	0.047	−0.122	(−0.075, 0.015)	0.505	−0.134*	(−0.089*, 0.018)	0.045

*p ≤ 0.05; **p ≤ 0.01; *r*, correlation coefficient; *β*, regression coefficient; partial *r*^2^ for independent variables. TAC, Total antioxidant capacity; MDA, Lipid peroxidation as malondialdehyde; SOD, Super oxide dismutase; GPx, Glutathione peroxidase enzyme; IL-10, Interleukin-10; TNF-*α*, Tumor necrosis factor-alpha; IGF-1, Insulin-like growth factor-1.

**Table 5:** Association between age and metabolic biomarkers before (R1) and at the end of Ramadan (R2) for PRE-M and POST-M healthy women (*n* = 62).

Independent variable/ dependent variable	PRE-M (*n* = 31)				POST-M (*n* = 31)			
	Age (years)							
	R1		R2		R1		R2	
	(β, SE)	*p*-value	(*β*, SE)	*p*-value	(*β*, SE)	*p*-value	(*β*, SE)	*p*-value
Estrogen	−0.001 ± 0.001	0.280	−0.050 ± 0.075	0.508	−0.019 ± 0.022	0.270	0.122 ± 0.110	0.772
Progesterone	−0.009 ± 0.004*	0.026	−0.071 ± 0.098	0.479	−0.017 ± 0.015	0.237	1.780 ± 1.040	0.564
TAC	−0.404 ± 0.555	0.474	−0.010 ± 0.053	0.859	−0.808 ± 0.446	0.959	0.110 ± 0.001	0.924
MDA	−0.135 ± 0.052*	0.017	−0.194 ± 0.110*	0.019	−0.089 ± 0.073	0.136	−0.444 ± 0.289	0.458
SOD	0.003 ± 0.018	0.875	0.013 ± 0.027	0.618	0.008 ± 0.034	0.849	−0.029 ± 0.011	0.487
GPx	−0.306 ± 0.218	0.174	−0.032 ± 0.030	0.312	−0.021 ± 0.011	0.950	−0.101 ± 0.001	0.989
IL-10	0.068 ± 0.025*	0.014	0.003 ± 0.013	0.828	0.054 ± 0.035*	0.048	0.014 ± 0.023	0.537
TNF-α	0.003 ± 0.028	0.909	0.014 ± 0.024	0.580	0.002 ± 0.043	0.826	0.043 ± 0.051	0.404
IGF-1	−0.221 ± 0.423	0.605	−0.166 ± 0.412	0.690	−0.100 ± 0.331*	0.046	−0.163 ± 0.351	0.047*

*p ≤ 0.05; **p ≤ 0.01; *r*, correlation coefficient; *β*, regression coefficient; partial SE = standard error for independent variables. TAC, Total antioxidant capacity; MDA, Lipid peroxidation as malondialdehyde; SOD, Super oxide dismutase; GPx, Glutathione peroxidase enzyme; IL-10, Interleukin-10; TNF-α, Tumor necrosis factor-alpha; IGF-1, Insulin-like growth factor-1.

## Discussion

The current study was designed to explore the effect of observing RIF on both pre-and post-menopausal healthy women in Saudi Arabia. This study unravels major differences between the two groups in terms of their hormonal and inflammatory responses to RIF. Interestingly, observing RIF was associated with improvements in some anthropometric, metabolic, inflammatory, and oxidative stress markers in both PRE-M and POST-M healthy women, with distinct differences in hormonal changes between the pre-and post-menopausal women.

### Anthropometric measures

The reported effect of fasting during Ramadan on body fat, weight, and BMI is consistent with the significant reduction in waist circumference observed in both women PRE-M and POST-M groups. Fat levels considerably decreased at the end of Ramadan, paralleling a significant reduction in waist circumference (34). This could result in a shift in the pattern of body fat redistribution after Ramadan. The reported significant decreases in FM and FMI in both groups at the end of the RIF period came despite the notion that FFM was unaffected by the RIF. In contrast, RIF-induced weight loss associated with decreased FFM has already been observed in other studies (19). However, a significant effect of RIF on FFM was not observed during this study. This is a crucial physiological finding because FFM plays a key role in blood glucose homeostasis, functional capacity, and resting energy expenditure (45). RIF could facilitate losing body weight and FM in some individuals. The adaptations acquired during fasting are transient and generally reversible within a short period (46). Thus, developing long-term body composition maintenance strategies at the end of Ramadan is important. Because glucose and fluids are less readily available to the body, stored body fat acts as a key substrate for energy production, resulting in lower fat content and body weight (19, 26). A study linked Ramadan fasting-related weight loss to a decrease in total calorie intake, as energy balance is critical in regulating changes in body weight (26). The current findings on body weight and anthropometric changes are consistent with the broad literature and are reported in more than one systematic review and meta-analysis on Ramadan fasting in healthy people (18, 34, 47).

### Glucose homeostasis, lipid profile, and blood pressure

Regarding the lipid profile, HDL-C significantly increased in both groups, especially in PRE-M women at the end of the RF period. TG significantly reduced in both groups, but the difference between the groups was not significant, whereas cholesterol and LDL-C remained unchanged, according to Kul et al. (47). In a study involving a healthy population, only women experienced a substantial increase in HDL-C levels. Factors including dietary habits, physical characteristics, type of fat saturation, fat percentage, simple sugars percentage in diet, and weight loss could affect the lipid profile. Furthermore, Kul et al. (47) found that participants’ metabolic markers and body weight were altered during Ramadan fasting compared to pre-fasting values. These findings are compatible with the current study’s findings since Ramadan fasting is different from other fasting regimens in that it entirely prohibits the ingestion of solid meals, liquids and water, which could impact blood sample concentration. Ramadan also significantly affects people’s behavioral habits and lifestyles, such as shortened sleeping hours and patience. As a result, Ramadan fasting is distinct from alternate-day fasting. According to Osman et al. (48), fasting during Ramadan has minor benefits for body composition by reducing body mass in both healthy and obese individuals, and the consequences are generally temporary and varied. However, there is a more consistent improvement in blood lipid profile during Ramadan fasting, and this often lasts beyond Ramadan time.

The altered sleep–wake cycle influences food and fluid consumption, which regulates energy intake and expenditure throughout the Ramadan fasting period (49). As a result, at the end of RIF, glucose levels in both groups were stable in this study. Moreover, during Ramadan, altered sleep patterns and psychological/social activities may change the cyclical pattern of numerous hormonal variables related to energy intake regulation and energy metabolism (49). Both groups’ systolic blood pressure remained stable; however, diastolic blood pressure drastically changed. The reduction was greater in POST-M women than in PRE-M women. Lower blood pressure during RIF could be attributed to a variety of causes, including weight loss, age, and reduced catecholamine synthesis. These factors cause a decrease in sympathetic tone, which results in a reduction of cardiac output, blood pressure, and heart rate (21, 50). The present study showed that PRE-M women had significantly lower estrogen levels than POST-M women, although progesterone levels remained constant in both groups. Several investigations have produced contradictory findings about the effect of RF on reproductive hormones in healthy women (3, 4, 51). The current findings on lipid profile and cardiometabolic risk factors are also in line with the established findings reported in systematic reviews and meta-analyses on RIF in healthy people (21, 47).

### Inflammatory and oxidative stress markers

Because of the differences in hormonal secretions between the two groups, RF has different health impacts on PRE-M and POST-M women. SOD is an antioxidant enzyme that catalyzes the dismutation of two superoxide anions (*O_2_) molecules to hydrogen peroxide (H_2_O_2_) and molecular oxygen (O_2_) to help counteract the oxidative stress of superoxide anions (52). SOD levels were significantly greater within each group (PRE-M and POST-M); however, there was no significant difference between the two groups at the end of the RIF. However, GPx levels increased in both groups, with PRE-M women having greater levels of both GPx and IL-10. GPx is a cytosolic enzyme that catalyzes the conversion of hydrogen peroxide to water and then the conversion of peroxide radicals to oxygen and alcohol, which minimizes the risk of superoxide anion (53).

TAC and MDA biomarkers are used to assess oxidative stress, whereas IGF-1 serves as an aging biomarker. These biomarkers did not change in both groups (PRE-M and POST-M). MDA is an end product of lipid peroxidation that participates in lipid radical formation and oxygen uptake in animal tissues as an oxidative stress mechanism initiated by reactive oxygen species. MDA acts as an endogenous lipid peroxidation biomarker and is considered an important oxidative stress biomarker (54, 55). The results indicated reduced oxidative stress during RF in both groups that are facilitated by increased SOD levels and unchanged MDA levels. A significant increase in IL-10 and a decrease in TNF-*α* revealed an enhanced inflammatory response in PRE-M women as compared to POST-M women.

The decrease in cytokine levels during RF could also be attributed to reduced oxidative stress and reactive oxygen species, which participate in the activation of the transcriptional factor responsible for the expression of pro-inflammatory cytokines such as TNF-α (56). The reduction in C-reactive protein indicated significant suppression of inflammatory biomarkers in PRE-M women in comparison to POST-M women. This might be because of the substantial decrease in inflammatory biomarkers (C-reactive protein, IL-6) during IF (57). The reported results on the inflammatory and oxidative stress markers are consistent with the current literature supporting the beneficial effect of observing RIF (19, 24, 40).

### Relationship between anthropometrics and age with cytokines and oxidative stress biomarkers

The relationship between BMI, percent body fat, and age as independent variables, and sex hormones and metabolic biomarkers as dependent variables revealed that as BMI increased, the estrogen levels also increased in POST-M women. In contrast, progesterone levels decreased significantly before and after Ramadan. However, an increase in the percentage of body fat boosted estrogen levels in POST-M women while lowering progesterone levels in both groups. MDA reduced as percent body fat dropped in POST-M women, indicating lower oxidative stress in these women. The BMI of POST-M women and the percent body fat of both groups increased TNF-*α*, which could be an indication of inflammation. During Ramadan, both BMI and percent body fat had a negative influence on IGF-1, resulting in reduced bone density, less muscle mass, and changed lipid levels (48). Ramadan fasting did not affect women’s sex hormones, regardless of age. However, before Ramadan, progesterone levels in PRE-M women decreased significantly with age. According to these findings, age had a negative impact on female sex hormones before Ramadan. This finding is consistent with those reported in other relevant studies (3, 4, 51). The strong negative connection between PRE-M women’s age and lipid MDA before and after Ramadan could be because lipid MDA levels decrease with age, which may contribute to lower oxidative stress. The fundamental metric for evaluating the current potential of oxidative stress in aging and other age-related disorders is antioxidant capacity. The first step in anticipating oxidative stress in the aging process is to estimate the reducing power/antioxidant capacity since an imbalance between antioxidants and oxidants causes oxidative stress (58). Abbasi et al. reported that in subjects under a high-fat diet, both high-intensity interval training and IF may help to improve lipid profile (59). Still, their combination may have little synergistic effect. Before Ramadan, a positive association was found between the age of both groups and IL-10, indicating that the level of IL-10 increased with age, implying that the women will be protected against inflammation because IL-10 inhibits the activity of Th1 cells, natural killer cells, and macrophages during infection (60). The negative relationship between POST-M women’s age and IGF-1 before and after Ramadan suggested that the hormone declined with age in POST-M women, resulting in low bone density, less muscular mass, and changed lipid levels (61).

The aforementioned findings of the current research are consistent with the broad literature supporting the health-improving effect of observing RIF by healthy people, particularly in terms of the improvements in cardiometabolic profile including anthropometric measures (BMI, body fatness, and waist circumference), lipid profile (increased HDL and reduced LDL, TG, and TC), and inflammatory and oxidative stress markers (increased anti-inflammatory IL-10, antioxidant SOD, GPx, decreased proinflammatory IL-6, TNF-*α*).

### Study strengths and limitations

The strength of our study is that we examined both pre-menopausal and post-menopausal women from the same living community to ensure homogeneity and avoid inequalities. Extensive questionnaires, physical measurements, and laboratory tests were employed to quickly identify the variables impacting the patients’ physiological and metabolic biomarkers before and after RIF. It is generally established that physiological and metabolic biomarkers differ significantly between the two groups; nevertheless, our findings show that the presence of RIF exacerbates this. Also, our findings suggest that RIF is associated with promoting positive metabolic alterations in both group states. Furthermore, variations in subjects’ commitment to the diet or IF schedule, also referred to as compliance or adherence, are a problem for nutrition research. However, unlike studies done in non-Islamic nations, where fasting systems can be broken or violated in any way, our study was carried out in an Islamic country, where everyone follows the system strictly from 4 a.m. Fajr (dawn) until 6 p.m. Maghrib (sunset), on average, for a full month. This indicates that the study’s strengths include its current research location and the caliber of its time-bound participants during the IF phase. However, the small sample size was the main limitation of this study, as it focused on a specific region in Saudi Arabia. In addition, all the participants were drawn from the same medium-income living community to ensure sample homogeneity and to avoid disparities and confounding socioeconomic and cultural factors. The non-fasting control group was excluded because it was difficult to find the same number of non-fasting Ramadan participants with similar criteria of fasting participants, and different nations varied in dietary and physiological habits as well. Measurements of women who are exempt from fasting during menstruation were taken on fasting days. Future research on the effects of RF on women with thyroid disease is required as this syndrome has become a public health concern: exploring fasting and peri-menopause will provide insight and valuable data; also, the effect of fasting on neurotransmitters are excellent topic to venture into; furthermore, the impact of fasting on autophagy, stem cell activation, and DNA telomere attrition delay are excellent topics to venture into; pursuing research in this field is cutting-edge, with a significant scientific contribution to postponing aging and increasing longevity.

## Conclusion

It can be concluded that observing RIF is associated with reduced body weight, waist circumference, BMI, PBF, FM, FMI, TG, and DBP without affecting the other parameters. Furthermore, RIF might effectively help reduce inflammatory processes. This phenomenon was observed in PRE-M women with a significantly lower level of proinflammatory cytokine (TNF-*α*) and a substantially higher level of an anti-inflammatory biomarker (IL-10) as compared to POST-M women. The effect of RIF on cytokines provides biologically reasonable mechanisms regarding the beneficial aspects of fasting on lipid and carbohydrate balance, and autoimmune diseases. BMI and PBF changes were found to influence the cytokines and metabolic biomarkers, especially in POST-M women. Some metabolic biomarkers were observed with age changes in sex hormones.

## Data Availability

The datasets presented in this study can be found in online repositories. The names of the repository/repositories and accession number(s) can be found in the article/supplementary material.
